# Identifying gene mutations of Chinese patients with polycystic kidney disease through targeted next‐generation sequencing technology

**DOI:** 10.1002/mgg3.720

**Published:** 2019-05-06

**Authors:** Tao Wang, Qinggang Li, Shunlai Shang, Guangrui Geng, Yuansheng Xie, Guangyan Cai, Xiangmei Chen

**Affiliations:** ^1^ Department of Nephrology Chinese PLA General Hospital, Medical School of Chinese PLA, Chinese PLA Institute of Nephrology, State Key Laboratory of Kidney Diseases, National Clinical Research Center for Kidney Diseases Beijing China

**Keywords:** gene mutations, next‐generation sequencing, PKD, targeted NGS panel

## Abstract

**Background:**

Polycystic kidney disease (PKD) is the most common hereditary kidney disease. The main mutational genes causing autosomal dominant polycystic kidney disease (ADPKD) are PKD1 and PKD2 as well as some rare pathogenic genes. Unilateral PKD is rare in clinics, and its association with gene mutations is unclear.

**Methods:**

Targeted next‐generation sequencing (NGS) was performed to detect the renal ciliopathy‐associated genes (targeted NGS panel including 63 genes) in PKD patients.

**Results:**

Forty‐eight PKD1 and PKD2 mutation sites were detected in 44 bilateral PKD patients, of which 48 were PKD1 mutation sites (87.5%) and six were PKD2 mutation sites (12.5%). All of which exhibited typical ADPKD. Furthermore, we detected HNF1B heterozygous mutations in three families. Although these three patients showed HNF1B heterozygous mutations, their clinical characteristics differed and showed phenotypic heterogeneity.

**Conclusions:**

Targeted NGS panel was helpful in detecting typical ADPKD patients and even in non‐typical PKD patients. Macromutation in HNF1B may lead to bilateral PKD. The 16 novel PKD gene mutation sites and two novel PKD2 gene mutation sites discovered in this study have some significance in genetic counseling for ADPKD patients, and increase the number of studied families and expand the mutation database of ADPKD.

## 1 INTRODUCTION

Polycystic kidney disease (PKD) is a group of monogenic disorders that result in renal cyst development. Based on genetic patterns, PKD can be classified into autosomal dominant polycystic kidney disease (ADPKD) and autosomal recessive polycystic kidney disease (Harris & Torres, 2009). ADPKD is the most prevalent monogenic hereditary kidney disorder and the most common monogenic disorder that causes end‐stage renal disease (ESRD) (Torres, Harris, & Pirson, 2007). Its major presentations are multiple renal cysts that result in enlarged and irregular shaped kidneys. Depending on geographical location, its incidence is approximately 1:400–4000 (Torres et al., 2007). ADPKD develops primarily from PKD1 gene [MIM#601313] mutations on chromosome 16 and/or the PKD2 gene [MIM#173910] on chromosome 4 (Listed, 1994; Mochizuki et al., 1996). Clinical data indicate that PKD1 and PKD2 mutations account for 85% and 15% of ADPKD patients, respectively (Rossetti et al., 2007). ADPKD is heterogeneous with regard to locus and allele heterogeneity and phenotypic variability. PKD is usually bilateral, while unilateral PKD is rare. Unilateral PKD typically refers to segmental cystic abnormalities in 1 kidney. At present, few studies exist on unilateral PKD, and its pathological examination results are the same as those of ADPKD (Gouldesbrough & Fleming, 1998). Over time, unilateral PKD can change to bilateral PKD and further cause ESRD (Punia, Mohan, Bal, & Bansal, 2005). The pathogenesis of unilateral PKD and whether it is associated with gene mutations are unclear.

Currently, most ADPKD patients have PKD1 and/or PKD2 mutations; however, these mutations remain undetected in some patients. To better understand whether other genes cause ADPKD, as well as their resulting phenotypes, and to understand whether unilateral PKD is associated with gene mutations, we performed gene sequencing on PKD patients. Targeted next‐generation sequencing (NGS) was performed to screen 63 renal ciliopathy‐associated gene mutations in these patients.

## 2 MATERIALS AND METHODS

### 2.1 Study design

Gene sequencing was performed on 47 patients using our developed kidney disease panel. Possible pathogenic mutation sites were detected by Sanger sequencing. For some patients who did not detect suspected pathogenic genes, we further sequenced all exons and screened for suspected mutant genes. In addition, blood samples provided by family members of patients were used for segregation analyses. The detected mutation sites were carefully compared with the ADPKD Mutation Database (http://pkdb.mayo.edu), HGMD Professional (https://www.qiagenbioinformatics.com/products/human‐gene‐mutation‐database/) and relevant literature, and the mutation site pathogenicity were analyzed.

### 2.2 Patients

Forty‐seven unrelated PKD patients treated at the Chinese People's Liberation Army General Hospital between 2016 and 2017 were enrolled. All patients were confirmed to have unilateral or bilateral PKD by abdominal computed tomography or color Doppler ultrasound and volunteered for gene detection. The ages at PKD confirmation in these patients were 3–58 years. Forty‐four patients had bilateral PKD, and three had unilateral PKD. Among 44 bilateral PKD patients, 30 had clear family histories of dominant inheritance; therefore, validation was performed on immediate family members of 34 patients (a total of 65 family members). The three unilateral PKD patients were all isolated cases with no family history of this disease, and their parents did not have consanguineous marriages. All subjects or their legal guardians signed informed consent for genetic testing. The genetic analysis was approved by the Ethics Committee of the People's Liberation Army General Hospital (China).

### 2.3 Targeted NGS panel

Target region capture and next‐generation human gene analysis technology were performed for ciliopathy‐associated gene region and bioinformatics analyses. A kidney disease‐associated gene analysis panel including 63 genes was developed (Table S1). Combined with data including disease history and imaging examination, patients with kidney diseases and urinary system abnormalities could be screened at an early stage to reduce the damage of chronic kidney disease and provide gene diagnosis basis for personalized drugs in patients. This panel included many disease‐associated gene analyses including PKD, renal tuberculosis, Joubert syndrome, Meckel syndrome, short rib‐polydactyly syndrome (asphyxiating thoracic dysplasia/Jeune's syndrome), Bardet‐Biedl syndrome, and cranioectodermal dysplasia.

Targeted NGS panel was used for genetic analyses. NGS was performed on the NextSeq 500 apparatus (Illumina). This program included five main steps. (a) Nucleic acid extraction was performed on sample DNA using a genomic DNA extraction reagent kit, and the level of DNA quality was identified per the standard in Table S2. If the sample grade was level D, the sample was disqualified, and blood samples were collected again for DNA extraction. (b) The genomic library was constructed. Sample DNA was sheared to the range of 100–700 bp using the Covaris shearing method. The terminus was repaired, and an “A” was added. The product was purified for PCR amplification of the gene library, and the quality was detected per the standard in Table S3. If the sample grade was level D, the sample was disqualified and blood samples were collected again per the above procedures. (c) The target genes were captured. GenCap^®^ Kidney disease gene capture probe (MyGenostics, China) and library DNA were hybridized under set conditions. Streptavidin‐modified magnetic beads were used to covalently bind to biotin‐labeled probes to capture target genes. Finally, a magnetic separator rack was used to adsorb magnetic beads that carried target genes. Target genes were then eluted, purified, and enriched. (d) The NextSeq 500 tabletop sequencer was used for large‐scale sequencing. (e) Data analyses were performed using BWA software (http://bio-bwa.sourceforge.net/), GATK software (http://www.broadinstitute. org/gsa/wiki/index.php/GATK_resource_bundle), and ANNOVAR software (http://www.broadinstitute.org/gsa/wiki/index.php/GATK_resource_bundle). After comparison with Homo sapiens (hg19), a mutation site that satisfied the following conditions was screened: (a) it was present in target regions; (b) it caused amino acid changes; (c) its mutation frequency in the local population was lower than 15%; and (d) if known in the databases, its minor allele frequency was below 1% for autosomal recessive inheritance and 0.2% for autosomal dominant transmission. All filtered variants were further analyzed using Alamut v.2.9.0 software (Interactive Biosoftware, La Rochelle, France) for predicting functional effects with SpliceSiteFinder, MaxEntScan, NNSPLICE, GeneSplicer, Human Splicing finder, Polyphen‐2, SIFT, MutationTaster, Align GVGD and UMD‐Predictor (Morais et al., 2017).

### 2.4 Whole exome sequencing

Genomic DNA was isolated from lymphocytes and subjected to exome capture using the SureSelect Human All ExonV6 human exome capture arrays (Agilent) followed by next generation sequencing on the NextSeq 500 tabletop sequencer. Data analyses were performed following the procedure of targeted NGS panel‐based analysis.

### 2.5 Sanger sequencing

After gene mutation sites were detected using NGS, they were validated using Sanger sequencing. In addition, blood samples provided by the patients’ family members were also validated by Sanger sequencing. Primers were designed using Primer software for PCR amplification. Next, capillary electrophoresis sequencing was performed using a 3130XL sequencer. When reference sequences were found, the reference sequences and raw data were analyzed using Mutation Surveyor software (https://softgenetics.com/mutationSurveyor.php).

### 2.6 Multiplex ligation‐dependent probe amplification

In order to confirm the presence of large gene rearrangements in the HNF1B gene we performed multiplex ligation‐dependent probe amplification (MLPA) using the MLPA kit P241 25R (HRC‐Holland, Amsterdam, Netherlands).

## 3 RESULTS

### 3.1 Genetic characterization

Among 44 bilateral PKD patients (Table 1), PKD1 heterozygous mutations were detected in 35 patients, PKD2 heterozygous mutations were detected in three patients, both PKD1 and PKD2 mutations were detected in three patients, one PKD1 heterozygous mutation and one PKHD1 (2‐point mutation sites) compound heterozygous mutation were detected in one patient, and HNF1B heterozygous mutations were detected in two patients. The MLPA results of P43 further confirmed the heterozygous deletion of exon 1‐9, that is, the complete HNF1B heterozygous deletion.(Figure S1) Among three unilateral PKD patients (Table 2), one HNF1B heterozygous mutation was detected in one patient, and no clear pathogenic gene mutations were detected in two patients. Whole exome sequencing (WES) was performed on patient P47, and the results showed that this patient had three heterozygous mutations in LYZ, FGA, and GLI3. Sanger sequencing validation was performed on the patient's father, who had a heterozygous mutation at the LYZ site but normal FGA and GLI3. Because a blood sample could not be collected from the patient's mother, Sanger validation was not performed. We do not think the heterozygous mutations detected in the autosomal recessive genes were significant in the disease's development and progression.

**Table 1:** The mutation sites in 44 bilateral PKD patients

Family No.	Mutated gene	Inheritance	Exon	Nucleotide change	Amino acid change	Status	Segregation tested	Reference	Clinical significance
(a) *PKD1 mutations*									
P1	PKD1	Dominant	exon45	c.12310A>C	p.Ile4104Leu	Het	Yes	This study	Likely neutral
	PKD1	Dominant	exon15	c.4340C>T	p.Ala1447Val	Het	Yes	Yu et al. (2011)	Likely neutral
P2	PKD1	Dominant	exon40	c.11372_11373insGA TTACGACGTTGGCTGGGAG AGTCCTCACAATGG	p.Gly3791fs	Het	Yes	PKDB	Definitely pathogenic
P3	PKD1	Dominant	exon23	c.A8471C	p.Gln2824Pro	Het	Yes	This study	Likely neutral
P4	PKD1	Dominant	exon18	c.7288C>T	p.Arg2430X	Het	Yes	Phakdeekitcharoen et al. (2000)	Definitely pathogenic
P5	PKD1	Dominant	exon13	c.3140C>A	p.Ser1047X	Het	Yes	This study	Likely pathogenic
P6	PKD1	Dominant	exon15	c.4306C>T	p.Arg1436X	Het	Yes	Garcia‐Gonzalez et al. (2007)	Definitely pathogenic
P7	PKD1	Dominant	exon36	c.10678G>A	p.Gly3560Arg	Het	No	Tsuchiya et al. (2001)	Likely neutral
P8	PKD1	Dominant	exon31	c.10081G>A	p.Gly3361Arg	Het	Yes	This study	Likely neutral
P9	PKD1	Dominant	exon6	c.1291C>T	p.Gln431X	Het	Yes	This study	Likely pathogenic
P10	PKD1	Dominant	exon21	c.7984C>T	p.Gln2662X	Het	Yes	PKDB	Definitely pathogenic
P11	PKD1	Dominant	exon16	c.6935C>T	p.Ala2312Val	Het	Yes	PKDB	Likely neutral
P12	PKD1	Dominant	exon28	c.9637T>G	p.Phe3213Val	Het	Yes	This study	Likely neutral
	PKHD1	Recessive	exon38	c.6245C>T	p.Thr2082Ile	Het	Yes		Likely neutral
	PKHD2	Recessive	exon32	c.4844C>T	p.Thr1615Met	Het	Yes		Likely neutral
P13	PKD1	Dominant	exon1	c.108dupC	p.Cys37Leufs*77	Het	No	Rossetti et al. (2012)	Definitely pathogenic
P14	PKD1	Dominant	exon23	c.8311G>A	p.Glu2771Lys	Het	Yes	Rossetti et al. (2000)	Likely pathogenic
P15	PKD1	Dominant	exon10	c.2039A>T	p.Tyr680Phe	Het	Yes	Liu et al. (2014)	Likely neutral
P16	PKD1	Dominant	exon15	c.4810G>A	p.Val1604Met	Het	Yes	Yu et al. (2011)	Likely neutral
P17	PKD1	Dominant	exon36	c.10678G>A	p.Gly3560Arg	Het	No	Tsuchiya et al. (2001)	Likely neutral
P18	PKD1	Dominant	exon23	c.8426_8428del	p.2809_2810del	Het	Yes	This study	Likely neutral
P19	PKD1	Dominant	exon15	c.5014_5015del	p.Arg1672Glyfs*9	Het	Yes	Watnick et al. (1999)	Definitely pathogenic
P20	PKD1	Dominant	exon15	c.6544C>T	p.Gln2182X	Het	Yes	This study	Likely pathogenic
P21	PKD1	Dominant	exon25	c.9136C>T	p.Arg3046Cys	Het	Yes	Liu et al. (2015)	Likely pathogenic
	PKD1	Dominant	exon15	c.6915+2T>G	splicing	Het	Yes	Perrichot et al. (2000)	Definitely pathogenic
P22	PKD1	Dominant	exon23	c.8464G>A	p.Val2822Met	Het	Yes	Hwang et al. (2016)	Likely neutral
P23	PKD1	Dominant	exon45	c.12142G>T	p.Val4048Leu	Het	Yes	This study	Likely neutral
	PKD1	Dominant	exon25	c.9157G>A	p.Ala3053Thr	Het	Yes	Chang et al. (2013)	Likely pathogenic
	PKD1	Dominant	exon10	c.2039A>T	p.Tyr680Phe	Het	Yes	Liu et al. (2014)	Likely neutral
P24	PKD1	Dominant	exon23	c.8464G>A	p.Val2822Met	Het	Yes	Hwang et al. (2016)	Likely neutral
	PKD1	Dominant	exon39	c.11258G>C	p.Arg3753Pro	Het	Yes	This study	Likely pathogenic
P25	PKD1	Dominant	exon26	c.9272T>G	p.Met3091Arg	Het	Yes	This study	Likely neutral
	PKD1	Dominant	exon11	c.2527T>C	p.Ser843Pro	Het	Yes	Yu et al. (2011)	Likely neutral
P26	PKD1	Dominant	exon44	c.12138+5G>A	splicing	Het	Yes	This study	Likely neutral
	PKD1	Dominant	exon36	c.10678G>A	p.Gly3560Arg	Het	Yes	Tsuchiya et al. (2001)	Likely neutral
P27	PKD1	Dominant	exon23	c.8295_8296insATCCTCATGCGC	p.Ser2766delinsILMRS	Het	Yes	Rossetti et al. (2000)	Definitely pathogenic
P28	PKD1	Dominant	exon40	c.11314delG	p.Ala3772Profs*54	Het	Yes	Liu et al. (2015)	Definitely pathogenic
P29	PKD1	Dominant	exon33	c.10321C>T	p.Gln3441X	Het	Yes	Obeidova et al. (2014)	Definitely pathogenic
P30	PKD1	Dominant	exon36	c.10678G>A	p.Gly3560Arg	Het	Yes	Tsuchiya et al. (2001)	Likely neutral
	PKD1	Dominant	exon43	c.11944C>T	p.Gln3982X	Het	Yes	Rossetti et al. (2007)	Definitely pathogenic
P31	PKD1	Dominant	exon7	c.1591G>A	p.Glu531K	Het	Yes	Hwang et al. (2016)	Likely neutral
P32	PKD1	Dominant	exon21	c.7985dupA	p.Gln2663Alafs*159	Het	Yes	This study	Likely pathogenic
P33	PKD1	Dominant	exon5	c.862C>T	p.Gln288X	Het	Yes	Bataille, Berland, Fontes, and Burtey (2011)	Definitely pathogenic
P34	PKD1	Dominant	exon15	c.3792C>A	p.Tyr1264X	Het	No	PKDB	Definitely pathogenic
P35	PKD1	Dominant	exon8	c.1722+1G>C	splicing	Het	No	Audrézet et al. (2012)	Definitely pathogenic
P36	PKD1	Dominant	exon18	c.7300C>T	p.Arg2434Trp	Het	Yes	Hoefele, Mayer, Scholz, and Klein (2011)	Likely pathogenic
(b) *PKD2 mutations*									
P37	PKD2	Dominant	exon4	c.1094+1G>A	splicing	Het	Yes	Chung et al. (2010)	Definitely pathogenic
P38	PKD2	Dominant	exon4	c.964C>G	p.Arg322Gly	Het	Yes	Audrézet et al. (2015)	Likely pathogenic
P39	PKD2	Dominant	exon5	c.1249C>T	p.Arg417X	Het	Yes	Pei et al. (1998)	Definitely pathogenic
(c) *PKD1&PKD2 mutations*									
P40	PKD1	Dominant	exon23	c.8444C>T	p.Ala2815Val	Het	No	Yu et al. (2011)	Likely neutral
	PKD1	Dominant	exon18	c.7480G>A	p.Glu2494Lys	Het	No	This study	Likely neutral
	PKD2	Dominant	exon6	c.1546G>T	p.Val516Leu	Het	No	Yu et al. (2011)	Likely neutral
P41	PKD2	Dominant	exon10	c.2051dupA	p.Tyr684_S685delinsX	Het	Yes	This study	Likely pathogenic
	PKD1	Dominant	exon37	c.10973A>G	p.Lys3658Arg	Het	Yes	This study	Likely neutral
P42	PKD2	Dominant	exon10	c.2083dupA	p.Ala696Sfs*2	Het	Yes	This study	Likely pathogenic
	PKD1	Dominant	exon36	c.10678G>A	p.Gly3560Arg	Het	Yes	Tsuchiya et al. (2001)	Likely neutral
(d) *HNF1B mutations*									
P43	HNF1B	Dominant	exon1−9	Complete deletion	Complete deletion	Het	No		
P44	HNF1B	Dominant	exon4	c.894_895delCT	p.Asn298Lysfs*21	Het	No		

**Table 2:** The mutation sites in three unilateral PKD patients

Family No.	Mutated gene	Inheritance	Exon	Nucleotide change	Amino acid change	Status	Mutation frequency in the local population	Clinical significance
P45	HNF1B	Dominant	exon1	c.313G>A	p.Glu105Lys	het	0.00217	Uncertain
	ZNF423	Recessive	exon4	c.2237A>G	p.Lys746Arg	het	0.0001	VUS
P46	ALMS1	Recessive	exon8	c.2351A>G	p.Flu784Gly	het	0.0068	VUS
	BBS2	Recessive	exon8	c.865A>G	p.Ile289Val	het	0.0174	VUS
	BBS9	Recessive	exon19	c.2086G>A	p.Asp696Asn	het	0.0068	VUS
	CSPP1	Recessive	exon27	c.3298T>C	p.Trp1100Arg	het	0.0308	VUS
	IFT122	Recessive	exon30	c.3686G>A	p.Arg1229His	het	0.0016	VUS
	TTC8	Recessive	exon14	c.1328G>A	p.Arg443Gln	het	—	VUS
P47	CEP290	Recessive	exon38	c.5127G>T	p.Gln1709His	het	—	VUS
	NPHP4	Recessive	exon10	c.1196A>G	p.Glu399Gly	het	0.0026	VUS
	PKHD1	Recessive	exon28	c.3179A>G	p.Asn1060Ser	het	—	VUS

Forty‐eight mutation sites were detected in the PKD1 and PKD2 genes (Table 1). Compared with PKDB, HGMD Professional and literature reports, we found a total of 18 novel variants (16 in PKD1 and two in PKD2). All mutation sites were analyzed for pathogenicity in strict accordance with the American College of Medical Genetics and Genomics guidelines. Sixteen definite pathogenic mutation types (14 PKD1 gene sites and two PKD2 gene sites) were detected in 16 probands. These mutations included eight nonsense, four frameshift, three splicing, and one insertion mutation. We speculated that the splicing mutation (c.1722+1G>C, splicing) in PKD1 was a novel pathogenic mutation. There were 12 likely pathogenic mutations (nine PKD1 and three PKD2 gene sites) in 12 probands. These mutations included four nonsense, two frameshift, and six missense mutations. We predicted seven novel, likely pathogenic mutations (five PKD1 and two PKD2 gene sites) (Table 1). There were 20 likely neutral mutations (19 PKD1 and 1 PKD2 gene site) in 20 probands. These mutations included 18 missense, one splicing, and one frameshift mutation. We predicted 10 novel, likely neutral mutations (10 PKD1 gene sites) (Table 1).

### 3.2 Clinical manifestation

Among 42 patients with bilateral PKD caused by PKD1 and/or PKD2 mutations, 11 (26.2%) had combined hepatic cysts or polycystic liver. Some had already progressed to ESRD and required renal replacement therapy. Two with HNF1B mutations had no combined hepatic cysts, and their kidney functions were within the normal range. P44 had type 2 diabetes mellitus, while P43 had no history of diabetes mellitus but had increased uric acid (blood uric acid: 553.5 µmol/L). The three patients with unilateral PKD had a left polycystic kidney with normal morphology of the right kidney. P45 had no history of diabetes mellitus but had increased blood creatinine and uric acid (blood creatinine: 122.6 µmol/L; blood uric acid: 613 µmol/L). This patient's father also had a heterozygous mutation at the same HNF1B gene site (Table 2); however, the father had normal bilateral kidney morphology with no history of diabetes mellitus. The other two unilateral PKD patients (P46 and P47) with no detected pathogenic genes had normal creatinine and no family history of cystic diseases.

## 4 DISCUSSION

NGS can target the whole genome for detection. It has the advantages of high resolution, high throughput, high efficiency, and high sensitivity. It can increase gene detection efficiency and reduce gene detection costs (Mardis, 2008). After mutation sites are detected by NGS, Sanger sequencing validation can further increase detection accuracy; however, NGS cannot detect deleted or repeated fragments in nucleic acid sequences. Therefore, for typical ADPKD patients whose gene mutations cannot be detected by NGS, MLPA should be used to detect whether PKD1 or PKD2 have deleted or repeated fragments to avoid missed diagnoses. Synonymous mutations are generally considered not to affect amino acid changes in proteins, However, if a silent mutation in exonic splicing enhancer sequences may affect the splicing of MRNA (Ramser et al., 2005). Therefore, we need to make relevant analysis and prediction.

**Figure 1:**
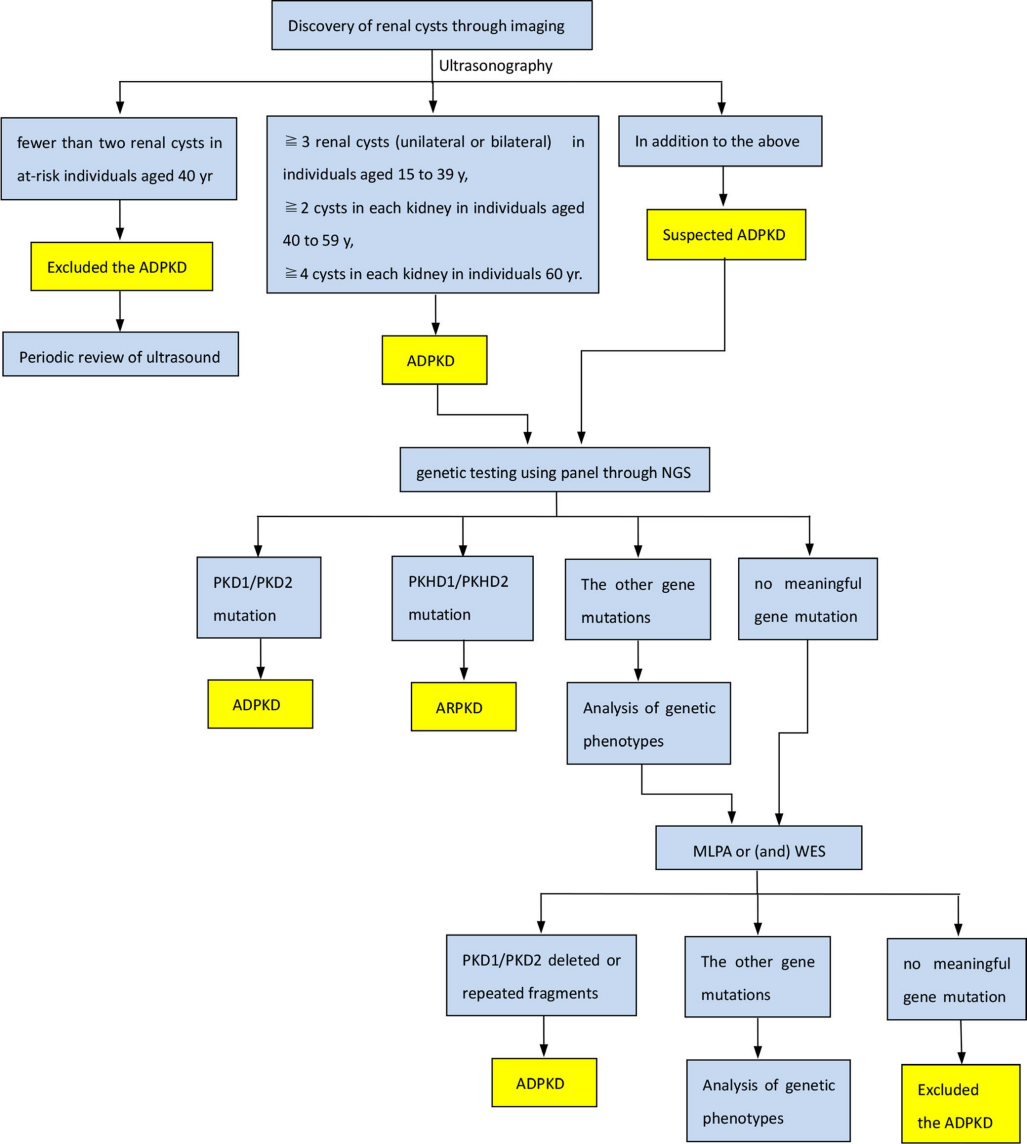
The diagnosis process for patients with a positive family history. ADPKD, autosomal dominant polycystic kidney disease; ARPKD, autosomal recessive polycystic kidney disease; MLPA, multiplex ligation‐dependent probe amplification; NGS, next‐generation sequencing; WES, whole exome sequencing

At present, diagnosis of ADPKD is based on family history and ultrasound imaging. In families of unknown genotype, the presence of three or more (unilateral or bilateral) renal cysts is sufficient for establishing the diagnosis in individuals aged 15–39 years, two or more cysts in each kidney is sufficient for individuals aged 40–59 years, and four or more cysts in each kidney is required for individuals 60 years (Pei et al., 2009). Which lead to a delay in or lack of diagnosis of ADPKD patients with no cysts in the kidneys and no apparent family history, which may result in inappropriate management. With the development of genetic testing technology, it is possible to make a definitive diagnosis before the onset age of a patient. Genotypes can also provide the basis for disease progression and prognosis (Jin et al., 2016). Based on our research and clinical experience, we designed the diagnosis process for patients with a positive family history (Figure 1).

Applying our developed targeted NGS panel detected a pathogenic gene that caused this disease in 44 bilateral PKD patients. Forty‐two patients had detected PKD1 and/or PKD2 gene mutations, and two patients had detected HNF1B heterozygous mutations. Therefore, performing mutation analyses on only PKD1 and PKD2 in ADPKD patients who require gene diagnosis is insufficient, and application of our gene detection panel or even WES and MLPA are necessary (MLPA is a novel diagnostic tool for genetic screening, which is gradually becoming the principal method for the detection of exon deletion and duplication (Schouten et al., 2002), as they can detect HNF1B, gene repetition and deletion, or other possible pathogenic gene mutations. Currently, PKDB describes 2323 PKD1 mutations and 278 PKD2 mutations. We detected 42 PKD1 mutation sites (87.5%) and six PKD2 mutation sites (12.5%), which were similar to percentages previously reported in the literature. Among 44 bilateral PKD patients, the PKD1 (p.G3560R) missense mutation was detected in five families. This site might be a neutral site. We speculated that this mutation site had greater prevalence in the Chinese population. The pathogenicity of the novel mutation sites discovered in this study must be confirmed in other families in future studies. We detected no large gene deletions or repetitions in this patient group, which might be due to the limitations of NGS. We discovered 16 novel PKD1 gene mutation sites and two novel PKD2 gene mutation sites in the Chinese population, which could enrich the ADPKD Mutation Database.

The onset of ADPKD caused by PKD1 and/or PKD2 gene mutations and the severity of their phenotypes are not only associated with gene mutations (germ cell mutations) but are also associated with somatic cell mutations or deletions in normal alleles caused by environmental factors such as toxins and infection (Feng, Watnick, Onuchic, & Germino, 1996). ADPKD caused by PKD1 and/or PKD2 mutations is usually bilateral. Its phenotypes are associated with patient gender, whether the patient has hypertension or had a urologic event (gross hematuria, flank pain, or cyst infection) before age 35, and gene mutation characteristics (genotypes)(Cornec‐Le et al., 2016; Jin et al., 2016). The average age of ERSD onset caused by PKD1 mutations is 54.3 years, while the average age of ERSD onset caused by PKD2 mutations is 74 years (Kurashige et al., 2015). Because the average age of this patient group was 39.4 years and most patients had not reached the above ages, most patients had normal kidney functions. We have not performed genotype‐phenotype analyses on these patients; however, we will continue to monitor them and further perform genotype‐phenotype analyses.

The HNF1B gene [MIM 189907] that causes the phenotype similar to polycystic kidneys is transcription factor 2 located on chromosome 17q12. HNF1B can directly regulate PKHD1 transcription. Inhibiting PKHD1 gene expression may result in human renal cyst formation (Hiesberger et al., 2004). HNF1B's effects on the kidneys may include renal cysts, solitary kidney, horseshoe kidney, renal dysplasia, and hydronephrosis (Clissold, Hamilton, Hattersley, Ellard, & Bingham, 2015). Renal cysts caused by HNF1B mutations are more heterogeneous; they can present as multiple, few, or no cysts, and some patients will enter into ESRD (Faguer et al., 2011). The severity of the HNF1B mutation‐associated kidney disease phenotype had no clear association with the genotype (Heidet et al., 2010). We detected HNF1B heterozygous mutations in three patients. Two patients had bilateral PKD, and their HNF1B mutations were both large mutations (P43 had a complete deletion and P44 had a frameshift mutation). We speculated that these 2 patients’ bilateral PKD diseases were caused by HNF1B mutations. P45 had unilateral PKD with a point mutation in HNF1B. We found that the gene mutation levels of these three patients might be related to the kidney phenotype, which was more severe in patients with large mutations and was inconsistent with previous study results. This difference may have been due to our fewer patients. After a larger number of patients with PKD caused by HNF1B mutations is increased, whether kidney phenotype is associated with genotype can be further analyzed. The association between HNF1B mutations and unilateral PKD remains unclear. Mutations in the HNF1B gene usually cause diabetes maturity‐onset diabetes of the young type 5 (MODY5) (Roehlen et al., 2018). Of the three patients with the HNF1B mutation we detected, P44 (32 years old) had type 2 diabetes, while P43 (56 years old) and P45 (39 years old) had no diabetes. The incidence of diabetes in these three patients did not show age‐related. The phenotype caused by HNF1B mutations is diverse and does not necessarily lead to the onset of diabetes (Chen et al., 2010). Even mutations at the same site show multiple phenotypes (Yorifuji et al., 2004). The father of P45 also had an HNF1B mutation, however, he did not have diabetes mellitus or renal structural abnormalities, and we have not functionally validated this mutation site. Therefore, the significance of this mutation site on renal cyst development is unclear. Further larger studies would be required to confirm whether HNF1B mutations are associated with unilateral PKD.

Unilateral PKD is rare, as are studies of it. Among the three cases of unilateral PKD discovered in this study, P45’s case may have been caused by an HNF1B mutation, while P46 and P47 had no gene mutations on the kidney disease panel and P47 had LYZ, FGA, and GLI3 heterozygous mutations on the WES. These three genes have not been reported to be associated with PKD in the past, and this patient had no clinical presentations associated with these three genotypes. Whether unilateral PKD is associated with the above genes requires further confirmation using further larger studies.

In conclusion, using our developed targeted NGS panel for gene detection is necessary for PKD patients. It can be used to confirm patient genotypes (with/without mutations, mutation numbers, and mutation types) and has important significance in confirming molecular diagnoses and predicting patient prognosis. Targeted NGS panel and WES on unilateral PKD patients are significant. Macromutation in HNF1B may lead to bilateral PKD. While the relationship between HNF1B and unilateral PKD needs further studies to confirm. We discovered 16 novel PKD1 gene mutation sites and two novel PKD2 gene mutation sites that can enrich the PKDB and are significant in genetic counseling for ADPKD patients, and the use of effective targeted NGS method in the molecular diagnosis of ADPKD will increase the number of studied families and expand the mutation database of ADPKD.

## CONFLICT OF INTEREST

The authors declare no conflict of interest.

## DATA AVAILABILITY STATEMENT

The datasets used and analyzed during the current study available from the corresponding author on reasonable request.
