# Supplementary figures and images for: Identifying gene mutations of Chinese patients with polycystic kidney disease through targeted next‐generation sequencing technology
Source: Mol Genet Genomic Med. 2019 May 6;7(6):e720. doi: 10.1002/mgg3.720 (PMC6565597; doi:10.1002/mgg3.720)

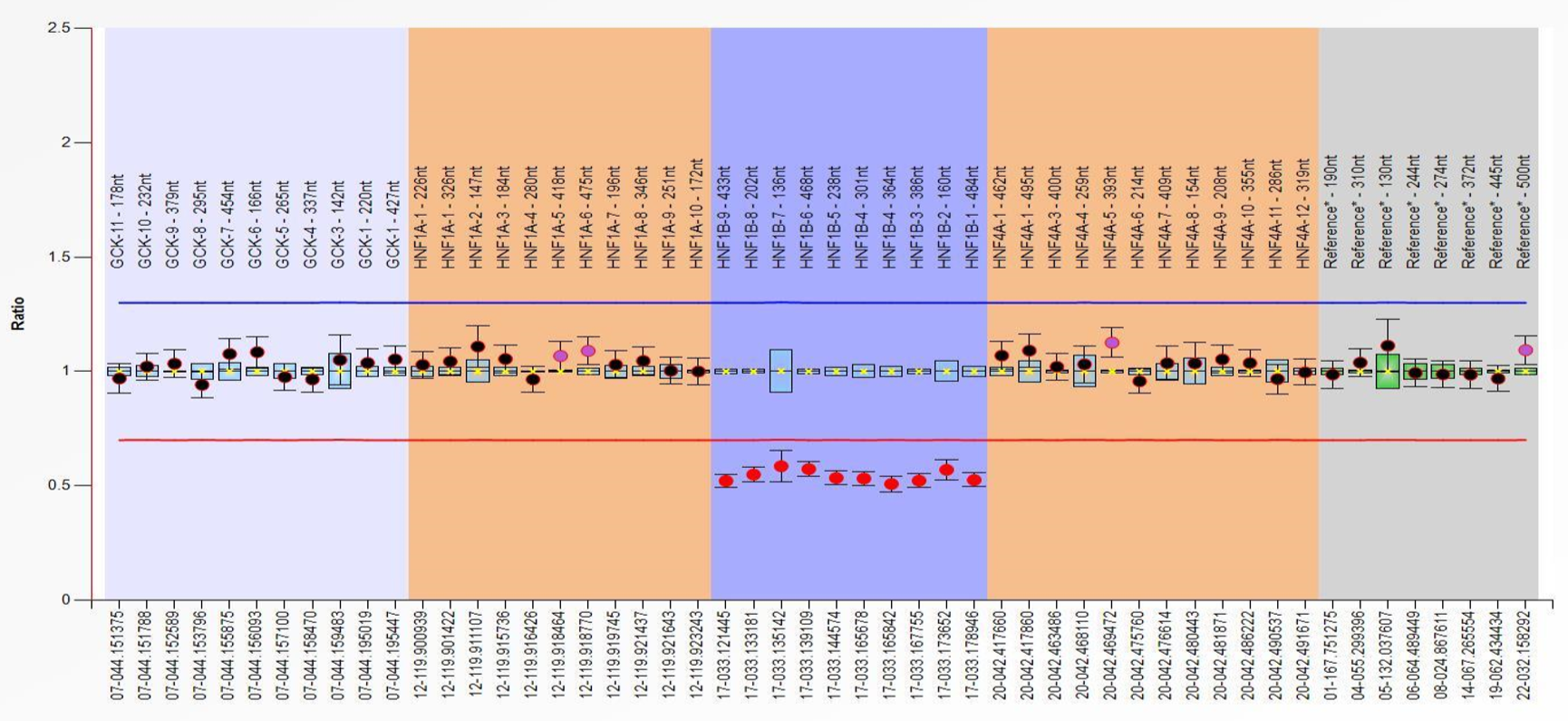

Supplement: Supplementary file 1 [file MGG3-7-e720-s001.tif]
