# Supplementary material for: Identifying gene mutations of Chinese patients with polycystic kidney disease through targeted next‐generation sequencing technology
Source: Mol Genet Genomic Med. 2019 May 6;7(6):e720. doi: 10.1002/mgg3.720 (PMC6565597; doi:10.1002/mgg3.720)
Supplement: Supplementary file 2 [file MGG3-7-e720-s002.doc]

| **Supplementary Table S1** Gene panel | | | | | | | |
| --- | --- | --- | --- | --- | --- | --- | --- |
| AHI1 | BBS12 | CEP290 | IFT43 | MUC1 | PKD1 | TMEM138 | UMOD |
| ALMS1 | BBS2 | CEP41 | IFT80 | NEK1 | PKD2 | TMEM216 | WDPCP |
| ANKS6 | BBS4 | CSPP1 | INPP5E | NEK8 | PKHD1 | TMEM231 | WDR19 |
| ARL6 | BBS5 | DYNC2H1 | INVS | NPHP1 | REN | TMEM237 | WDR34 |
| B9D1 | BBS7 | GLIS2 | IQCB1 | NPHP3 | RPGRIP1L | TMEM67 | WDR35 |
| B9D2 | BBS9 | IFT122 | LZTFL1 | NPHP4 | SDCCAG8 | TRIM32 | WDDR60 |
| BBS1 | CC2D2A | IFT140 | MKKS | OFD1 | TCTN2 | TTC21B | ZNF423 |
| BBS10 | CEP164 | IFT172 | MKS1 | PDE6D | TCTN3 | TTC8 |  |
