# Supplementary material for: Identifying gene mutations of Chinese patients with polycystic kidney disease through targeted next‐generation sequencing technology
Source: Mol Genet Genomic Med. 2019 May 6;7(6):e720. doi: 10.1002/mgg3.720 (PMC6565597; doi:10.1002/mgg3.720)
Supplement: Supplementary file 3 [file MGG3-7-e720-s003.doc]

| **Supplementary Table S2** The standard of DNA quality | | |
| --- | --- | --- |
| Concentration（ng/μl） | 260:280 | Grade level |
| >=30ng/μl | 1.7=<a=<2.0 | A |
| >=30ng/μl | 1.5<=a<1.7 OR 2.0<=a<2.3 | B |
| >=30ng/μl | <1.5 OR >2.3 | C |
| 20 -30 ng/μl | 1.7=<a=<2.0 | B |
| 20 -30 ng/μl | 1.5<=a<1.7 OR 2.0<=a<2.3 | C |
| 20 -30 ng/μl | <1.5 OR >2.3 | D |
| <20ng/μl | 1.7=<a=<2.0 | D |
| <20ng/μl | 1.5<=a<1.7 OR 2.0<=a<2.3 | D |
| <20ng/μl | <1.5 OR >2.3 | D |
