# Supplementary material for: Identifying gene mutations of Chinese patients with polycystic kidney disease through targeted next‐generation sequencing technology
Source: Mol Genet Genomic Med. 2019 May 6;7(6):e720. doi: 10.1002/mgg3.720 (PMC6565597; doi:10.1002/mgg3.720)
Supplement: Supplementary file 4 [file MGG3-7-e720-s004.doc]

| **Supplementary Table S3** The standard of gene library quality | | |
| --- | --- | --- |
| Concentration （ng/μl） | 260:280 | Sample grade |
| >=50ng/μl | 1.7=<a=<2.0 | A |
| >=50ng/μl | 1.5<=a<1.7 或 2.0<=a<2.3 | B |
| >=50ng/μl | <1.5或>2.3 | C |
| 30-50 ng/μl | 1.7=<a=<2.0 | B |
| 30-50 ng/μl | 1.5<=a<1.7或 2.0<=a<2.3 | C |
| 30-50 ng/μl | <1.5 或>2.3 | D |
| <30ng/μl | 1.7=<a=<2.0 | D |
| <30ng/μl | 1.5<=a<1.7或 2.0<=a<2.3 | D |
| <30ng/μl | <1.5 或>2.3 | D |

|  |
| --- |

|  |
| --- |
